# Small Signals Lead to Big Changes: The Potential of Peptide-Induced Resistance in Plants

**DOI:** 10.3390/jof9020265

**Published:** 2023-02-16

**Authors:** Julia Pastor-Fernández, Paloma Sánchez-Bel, Víctor Flors, Miguel Cerezo, Victoria Pastor

**Affiliations:** 1 Department of Biology, Biochemistry and Natural Sciences, School of Technology and Experimental Sciences, Universitat Jaume I, 12006 Castelló de la Plana, Spain; 2 Department of Plant Molecular Genetics, National Centre for Biotechnology, Consejo Superior de Investigaciones Científicas (CNB-CSIC), 28049 Madrid, Spain

**Keywords:** phytocytokines, induced resistance, priming, plant immunity, peptides

## Abstract

The plant immunity system is being revisited more and more and new elements and roles are attributed to participating in the response to biotic stress. The new terminology is also applied in an attempt to identify different players in the whole scenario of immunity: Phytocytokines are one of those elements that are gaining more attention due to the characteristics of processing and perception, showing they are part of a big family of compounds that can amplify the immune response. This review aims to highlight the latest findings on the role of phytocytokines in the whole immune response to biotic stress, including basal and adaptive immunity, and expose the complexity of their action in plant perception and signaling events.

## 1. The Plant Immune System

Plants are exposed to diverse types of stresses, both biotic and abiotic, and need to adjust their metabolism to respond rapidly to the changes that appear in their natural conditions. Plant cells harbor an array of receptors allowing the identification of the self and non-self [1,2]. Intracellular and cell-surface receptors work in combination to solve the identity of the threat and to activate immunity [3].

Non-self-molecules belong to a specific type of insect or microbe that are termed Pathogen/Microbe/Herbivore-Associated Molecular Patterns (PAMPs/MAMPs/HAMPs, respectively) [4]. These molecular signatures belong to bacteria, fungi, oomycetes, or arthropods and are often peptides, fatty acids, or oligosaccharides. 

In addition to non-self-recognition, pest or pathogen invasion also triggers the production and release of host-derived molecules. Pathogens and insects produce lytic enzymes to degrade plant tissues and access host cells leading to the release of degradation products. These molecules are commonly known as Damage-Associated Molecular Patterns (DAMPs) [5,6,7]. Both self and non-self-molecular patterns share common features in their recognition and signal transduction, such as recognition through specific Pattern Recognition Receptors (PRRs) in the cell surface and all the immune cascades events such as reactive oxygen species (ROS), the activation of mitogen-activated protein kinases (MAPKs), and the release of defensive hormones and genes. These responses are the hallmark of the so-called Pattern-Triggered Immunity (PTI) and Damage-Triggered Immunity (DTI), related to non-self and self-recognition, respectively. 

Recently, a more accurate classification for endogenous danger signals was proposed: (a) Those host molecules that are passively released after cell damage and disruption are primary endogenous danger signals and include “classical” DAMPs [2] such as cell wall fragments such as oligogalacturonides or cellulose fragments [8]; and (b) peptides that are produced actively by cells under biotic attack are secondary endogenous danger signals termed phytocytokines [2]. The production of phytocytokines often involves processing from a larger precursor that leads to the release of the mature peptide, which is perceived by neighboring cells to spread the danger alarm. Thus, unlike classical DAMPs, peptides may be present at the site of infection even if there is no cell disruption and they can also be released in adjacent intact cells [9]. 

## 2. Phytocytokines in Basal Immunity

Accumulating studies reveal the importance of small, secreted peptides in cell-to-cell signaling to coordinate cellular function including defense response in plants. Phytocytokines are small peptides secreted after damage perception that induce the amplification of immune responses in damaged and undamaged cells [2]. Tomato Systemin was the first signaling peptide found in plants [10]. Later, many peptides with a defense signaling function were identified in different plant species, such as PEPs (Plant Elicitor Peptides) from Arabidopsis, maize, and soybean [11,12,13]. Recently, there has been an emerging number of studies reporting the discovery of new peptides involved in plant defense against a variety of biotic stressors in different plant species (Table 1) [14,15,16,17]. 

Defense peptides are usually short in their amino acid chain. There are reported biologically active peptides ranging from 5aa in length such as phytosulfokine [36] to 67aa from the stable antimicrobial peptides biosynthesized by citrus in response to Huanglongbing disease [17], and they can be active at concentrations as low as femtomolar [38]. Regarding these mentioned features and their ubiquitous participation in plant physiological events and cell-to-cell communication, they have been considered by many authors as peptidic hormones [38,39], hence being suitable candidates for use as induced resistance (IR) elicitors.

The release of small defense peptides often involves the processing of a larger precursor propeptide, which differs in structure, indicating different processing mechanisms [40]. According to their precursor structure, there are peptides derived from precursors with an N-terminal secretion signal, from precursors not having an N-terminal secretion signal, and from proteins that have a different biological function [6,40]. The systemin precursor, ProSystemin, or the precursors of Arabidopsis PEPs, PROPEPS, are examples of proteins without an N-terminal secretion signal [10,11]. Recently, some research studies have shed light on the mechanism by which these two peptides are processed in plants. ProSystemin is hydrolyzed by subtilisins that release an inactive Systemin peptide that is further processed by a leucine aminopeptidase that removes the N terminal aa activating the functional peptide [41]. On the other hand, the Pep1 precursor, PROPEP1, is processed by Calcium-dependent metacaspases, which directly release the mature peptide [42]. Alternatively, Hydroxyproline-rich Systemins (HypSys) peptides derive from a precursor with an N-terminal secretion signal [31,33], and GmSubPep from soybean derives from a protein with a distinct primary function [36]. However, the processing mechanisms occurring to release HypSys and GmSubPep are poorly understood. In addition to proteolytic processing, some peptides require posttranslational modification to be biologically active and to interact with their receptor [43]. Posttranslational modifications include tyrosine sulfation, proline hydroxylation, and hydroxyproline arabinosylation [43,44]). Phytosulfokine (PSK) was the first identified peptide with posttranslational modifications, exhibiting sulfation at the two tyrosine residues [45]. Later, HypSys peptides were identified in tobacco and tomato as having proline hydroxylation [31,33]. 

Once the mature peptide is released, it triggers a cascade of signaling events and defense responses upon its perception by a membrane receptor. Peptides’ perception, signal transduction, and triggered defense responses are reviewed in the following sections.

### 2.1. Peptides’ Perception and Signal Transduction

A fast and efficient perception of plant surroundings is indispensable for plant survival. Similar to classical DAMPs or PAMPs, phytocytokines perception by membrane receptors of damaged and adjacent cells is crucial to ensure danger alarm spread leading to the amplification of immune signaling in undamaged tissues and resistance to pests and pathogens.

As other danger signals, plant defense peptides are perceived by membrane receptors that are usually receptor-like kinases (RLKs) with an extracellular domain that binds the peptide ligand, a transmembrane domain, and an intracellular kinase domain that ensures the initiation of an intracellular signaling cascade [46]. An increasing number of peptide–receptor pairs have been discovered in the last few years (Table 1). For instance, Arabidopsis Plant elicitor peptide 1 (Pep1) is perceived either by PEP RECEPTOR 1 (PEPR1) or 2 (PEPR2) whereas Arabidopsis Pathogen induced peptide 1 (PIP1) is perceived by RLK7 [14,47]. Furthermore, the receptor MALE DISCOVERER 1-INTERACTING RECEPTOR-LIKE KINASE2 (MIK2) interacts with SERINE-RICH ENDOGENOUS PEPTIDEs (SCOOPs) present both in plants and in pathogenic fungi and bacteria [21]. Additionally, very recently, RLK7 was also identified as a receptor of C-TERMINAL ENCODED PEPTIDE 4 (CEP4) [27]. These cell surface receptors often form complexes with coreceptors that enable the activation of downstream signaling upon ligand perception [1]. Several examples have been reported in the literature. The receptor-like Kinase BRI1-associated receptor Kinase (BAK1) functions as a coreceptor of multiple PRRs including those perceiving phytocytokines (Table 1). In addition, some receptor-like cytoplasmatic kinases such as Botrytis-induced kinase (BIK1) interact with PRR complexes to initiate the signal transduction upon complex activation in response to danger signals [48,49]. Both PEPR1 and RLK7 form a complex with BAK1, although early signaling triggered by PIP1 is only partially dependent on BAK1 [14,49]. Similarly, PEPR1 can directly phosphorylate BIK1, without relying on BAK1 [49]. However, PIP1 signal transduction was demonstrated to be BIK1-independent [14]. Upon recognition of the several SCOOPs peptide, MIK2 is also associated with BAK1 and its close homolog SERK4 and relies on BIK1 and PBL1 for downstream signaling events [20,21,50]. 

Remarkably, although BAK1 associates with multiple PRRs upon danger perception enabling signal transduction, it has been shown that pathogens induce BAK1 depletion, hijacking PTI responses [51]. When this happens, it has been demonstrated that the PEPR pathway ensures basal resistance inducing cell death and salicylate-related defenses [52]. This suggests that phytocytokines-triggered immune responses can also occur independently of common PTI signaling actors.

On the other hand, tomato Systemin is perceived by both LRR-RK receptors SYR1 and SYR2, which bind Systemin with high and low affinity, respectively, although more research is needed to confirm the binding mechanism [53]. The PEPR tomato ortholog PORK1 is also necessary to trigger Systemin-induced signaling since plants with silenced PORK1 but intact SYRs lack some Systemin responses [54]. However, it has not been demonstrated if PORK1 binds directly to Systemin or may function as a coreceptor of SYRs similar to the Arabidopsis receptor protein complexes mentioned above. Interestingly some peptides can be perceived by more than one receptor, generating different signals according to the peptide–receptor pair. 

Although many peptide–receptor complex pairs have been elucidated in the past few years, there are still many phytocytokines in which perception is still elusive. For instance, how maize ZmPeps and Zip1, soybean Peps, tomato CAPE1, or MaSAMP are perceived is still unknown (Table 1). Further research is needed to address this issue and improve our knowledge of phytocytokines’ perceptions and signal transduction. Techniques and methods to find new peptide ligand–receptor pairs are extensively reviewed elsewhere [38,55].

### 2.2. Peptides’ Perception and Signal Transduction

**Figure 1:**
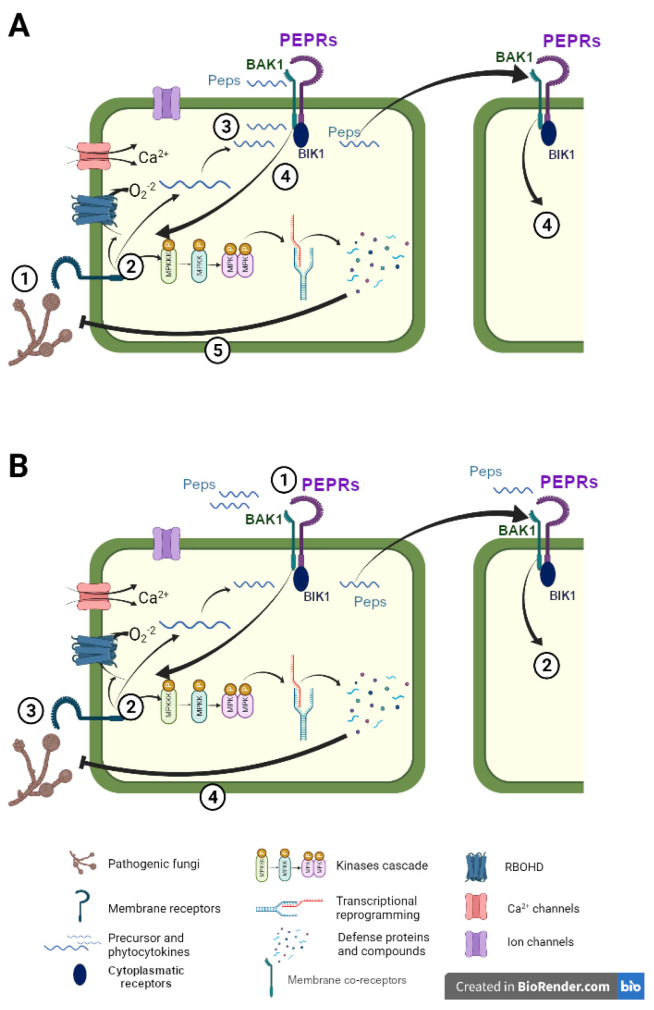
The natural function of defense peptides vs. Peptide-Induced Resistance. Example with the perception of the peptide Pep1 (**A**) Cellular responses against a pathogenic fungi infection. 1, Pathogen penetration in the cell is perceived by plant membrane receptors. 2, Intracellular signaling and defense responses are produced, including ROS production, the opening of ion and Ca^2+^ channels, and MAPK cascade activation that lead to downstream transcriptional reprogramming and defense compounds production. In parallel, peptide precursors are synthesized and phytocytokines are released. 3, mature peptides are released to the apoplast where they are perceived by damaged and adjacent cells. 4, phytocytokines trigger the amplification of defense responses. 5, the battery of defensive elements impairs pathogen success. (**B**) Mechanisms of Peptide-IR. 1, cell membrane receptors perceive the exogenously applied phytocytokine. 2, Immune responses are activated as in (**A**). 3, an invading fungal pathogen is perceived. 4, the plant is already prepared to counteract the infection, displaying a faster and stronger defense response that leads to enhanced resistance.

The binding of phytocytokines to their receptor triggers a cascade of defense signaling that leads to an amplification of the plant immune system to mount a defense response against invading attackers (Figure 1A). Defense peptides share common intracellular signaling elements with other self- and non-self-defense elicitors (Table 1). Although there is specific recognition of peptides by PRRs, triggered defense responses and intracellular signaling often overlap as it happens in response to PAMPs [4]. Resistance inducers and priming agents also trigger typical PTI defense responses, and primed plants have a potentiated defense in response to a challenge [56]. The next sections present the most common defense responses that are triggered by plant defense peptides and their natural role against biotic stresses.

#### 2.2.1. Increment of Cytosolic Ca^2+^

An increase in cytosolic Ca^2+^ is one of the earlier responses triggered by some phytocytokines and by other PAMPs and DAMPs during PTI, occurring within a few minutes, or even seconds after perception, upstream of subsequent immune responses [4]. Systemin perception triggers an increase in the intracellular calcium in mesophyll cells [57]. Similarly, Pep1, Pep3, SCOOPs, and CEP4 treatment produce an augmentation of cytosolic calcium in Arabidopsis [9,20,27,58]. Remarkably, it was recently reported that the release of Pep1 from PROPEP1 processing is catalyzed by Ca^2+^-dependent metacaspases [42,59]. On the other hand, PSK in tomatoes not only induces a Ca^2+^ increase but also Ca^2+^-dependent auxin responses for protection against *Botrytis cinerea* [35]. These findings suggest the importance of cytosolic Ca^2+^ in phytocytokines-triggered defense signaling.

#### 2.2.2. Effect on Ion Channels and Extracellular pH

The opening of ion channels and extracellular alkalinization is a hallmark response occurring after peptide treatment (Figure 1). Media alkalinization also occurs very rapidly (1 min) upon Flg22 or Elf18 treatment [60]. Rapid alkalinization factors (RALFs) peptides owe their name to their ability to alkalinize the extracellular media when applied to a cell suspension culture [33]. Similarly, tobacco and tomato HypSys, as well as peptides from soybean (GmSubPep, GmPep914, and GmPep890), also induce extracellular alkalinization when supplied to suspension-cultured cells (Table 1) [13,31,33,36]. In addition, the opening of ion channels by the modulation of plasma membrane H+ATPase activity is a Systemin-triggered early event [61].

#### 2.2.3. Production of Reactive Oxygen Species (ROS) and Activation of Mitogen-Activated Protein Kinases (MAPK)

ROS production is another cellular response to pathogen recognition and it mediates other defense responses in the plant [62,63]. PAMPs’, defense elicitors’, and many phytocytokines’ perceptions produce an increase in the oxidative burst (Table 1). In Arabidopsis, exogenous foliar application of PEP1, as well as PIP1, causes the production of H_2_O_2_ [11,14]. Pep3 induces both H_2_O_2_ and NO production, which are essential for functional Pep3-triggered immunity against *Pst*DC3000 since it is compromised in *rbohD/F* and *noa1* mutants [9]. Similarly, SCOOP12 induces ROS and Phosphatidic acid (PA) in Arabidopsis, suggested to be involved in ROS production, MAPK activation, and defense gene induction [16,64]. In addition, in Arabidopsis, the GRIM REAPER peptide (GRI) was shown to regulate ROS-dependent cell death [25,26]. In tomato, both CAPE1 and Systemin treatment trigger H_2_O_2_ formation [15,53] whereas in potato plants, HypSys also elicits H_2_O_2_ generation [32]. Conversely, RALF23 is a negative regulator of PAMP-induced ROS [23].

The activation of protein kinase cascades is a hallmark of PTI responses. MAPK cascades are essential signaling elements to ensure the defense signaling activation of downstream pattern recognition receptor complexes [4]. PIP1, SCOOPs, CEP4, Systemin, and Hypsys peptides induce MAPK activation in their respective species of origin [14,22,27,33,65]. Additionally, Systemin primes MPK3 and MPK6 phosphorylation upon *Plectosphaerella cucumerina* infection in *Arabidopsis thaliana* [66]. Parallelly, Calcium-dependent protein kinases (CDPKs), which are Ca^2+^ sensor protein kinases, are also activated upon several danger signals’ perception and trigger downstream defense responses [67]. However, very little is known about their implication in peptide-activated defenses. In this regard, Pep3 induction of MPK3 and WRKY33 and Pep-triggered immunity against *Pst*DC3000 is CDPK-dependent since it is impaired in the cpk mutants or when a kinase inhibitor is applied [9]. 

#### 2.2.4. Expression of Defense-Related Genes and Protease Inhibitors

Most phytocytokines induce the expression of a variety of defense-related genes in different plant species (Table 1). Although there are peptide-specific transcriptomic fingerprints, transcriptional changes triggered by defense peptides often overlap. In Arabidopsis, treatment with Peps induced the expression of plant defensin PDF1.2, MPK3, and WRKY33 transcription factor [9,11]. PIP1 treatment induces immune-related Flg22-INDUCED RECEPTOR KINASE1 (FRK1), the transcription factors WRKY30, WRKY33, and WRKY53 gene expression and expression of pathogen-related PR1 in protoplasts, and the transcription factor MYB51 in roots [14]. As PIP1, SCOOPs also induced FRK1, WRKY30/33 gene expression, and CYP81F2, involved in glucosinolate metabolism and resistance to fungi [16,21]. Similarly, it was recently observed that CEP4 also triggered the expression of the PTI marker gene FRK1 in Arabidopsis [27]. Systemin treatment induces the expression of defense-related genes, especially genes involved in the synthesis of JA, such as AOS and JA marker genes PI-I and PI-II [68]. Similar to Systemin, HypSys peptides activate the expression of octadecanoid pathway genes and essential pathogen- and herbivore-related genes [32]. CAPE1 activates the expression of pathogen-related genes PR1b, BETA-1,3-GLUCANASE (PR2), CYS PROTEASE (PR7), a chitinase, ETHYLENE RESPONSE FACTOR5 (ERF5), and AvrPto-DEPENDENT Pto-INTERACTING PROTEIN3 (Adi3) among others [15]. In soybean, GmSubPep, GmPep914, and GmPep890 peptides induce CYP93A1 gene expression, involving the synthesis of a phytoalexin, a chitinase, and chalcone synthase gene expression [13,36]. In Maize ZmPep1 induces some defense genes’ activation encoding for defense proteins, which includes *endochitinase A*, *PR-4*, *PRms*, and *SerPIN*, and a gene involved in the biosynthesis of the phytoalexin benzoxazinoid [12]. On the other hand, ZmPep3 increases the expression of indole biosynthetic genes together with genes encoding proteins associated with herbivory defense and biosynthetic enzymes for the production of volatile terpenes and benzoxazinoids [19]. Furthermore, in maize, Zip1 induces the expression of SA and JA marker genes and other defense-related genes such as WRKY transcription factors [37]. A new class of peptides named “stable microbial peptides” (SAMPs) were identified in some citrus hybrids tolerant to Huanglongbing (HLB) disease caused by the bacterial pathogen *Candidatus liberibacter asiaticus*. This peptide induces the expression of several defense-related genes, including pathogen-related PR1 and PR2 and phenylalanine ammonia-lyase 1 (PAL), involved in SA and phenylpropanoid biosynthesis through a pathogenesis-related gene1 (NPR1) and suppressor of G2 allele of skp1 (SGT1)-dependent manner [17].

On the other hand, some released peptides participate in positive feedback inducing the expression of their precursors. This is the case of AtPep1, which activates the expression of PROPEP1 [11]. PEPR activation also mediates PROPEP2/PROPEP3 activation (Yamaguchi & Huffaker, 2011). SCOOP12 and Pep1 trigger the expression of PROSCOOPS [16]. Similarly, CAPE1 induces the expression of its precursor protein PR1b [15] and Systemin induces the expression of ProSystemin [68]. An additional example shows that the Zip1 maize peptide induces the activity of the proteases that process its precursor PROZIP1 [37]. These findings suggest that a likely biological function of the positive feedback loop is to amplify defense responses improving resistance efficiency. 

A very common response triggered by defense peptides in tomato and other solanaceous species is the induction of Protease inhibitors (PIs). PIs inhibit insect digestive enzymes, making them key elements in plant defense against herbivory [69]. In fact, Systemin was identified when looking for signals that induced PI accumulation in tomato. Later, it was reported that Systemin is also present in potato, pepper, and nightshade where it also induces the accumulation of PIs. Similarly, HypSys found in tobacco, tomato, and potato can also trigger PIs against insects [32]. On the other hand, CAPE,1 a tomato peptide embedded in PR1b, was found to induce the expression of PIs [15]. In addition, the induction of PIs’ biosynthetic genes was also observed in maize due to ZmPep3 treatment [19].

#### 2.2.5. Hormonal following Phytocytokine Perception

Phytohormones are well known for their implication in plant defense, and their production in plants under attack is a conserved response across species. SA, ET, JA, and ABA are the main hormones regulating many resistance responses associated with basal immunity. as well as gene-for-gene and systemic resistance. In the literature, there are some examples of phytohormonal production upon defense peptide perception. In Arabidopsis, Pep1, SCOOPs, and CEP4 induce the accumulation of ET in Arabidopsis [16,27]. In maize, both ZmPep1 and ZmPep3 induce JA and ET [12,19], whereas Zip1 was observed to induce both JA and SA marker gene expression and strongly induce SA accumulation [37]. In Solanaceous species, Systemin induces the release of linolenic acid that leads to the production of JA and JA-Ile, as well as the biosynthesis of ET [53,70], and HypSys from tomato and potato were reported to activate the octadecanoid pathway and the production of JA [31,32]. In contrast, CAPE1 significantly induces SA accumulation in tomato [15]. In addition, other peptides seem to be involved in hormonal regulation upon different stresses. In fact, Arabidopsis PLANT NATRIURETIC PEPTIDE A, PNP-A, was shown to antagonize SA-mediated responses [22]. Similarly, the GRIM REAPER peptide was shown to be involved in hormonal regulation since SA and JA accumulation upon stress induced by O3 exposure was strongly reduced in gri knock-out plants [25]. On the other hand, PSK induces IAA and auxin-dependent responses in tomato plants against *Botrytis cinerea* infection [35]. Less evidence is reported regarding the role of ABA in these interactions in biotic stress. The cytoplasmatic complex RALF-FERONIA regulates several metabolic defensive pathways, including auxins, JA, ET, and ABA. More specifically, the perception of the peptide RALF1 by FERONIA inhibits the ABA signaling under salt stress by avoiding the opening of stomata. Nevertheless, stomata movement is essential for dealing with the entrance of pathogens, thus the RALF-FERONIA combination likely has a relevant influence on modulating immune responses [71,72]. 

#### 2.2.6. Other Basal Inducible Defense Responses Triggered by Phtocytokines

Among the inducible downstream defense responses, we found a few reports of peptides inducing callose accumulation. Callose is a β-1,3 glucan polymer that accumulates in the plant cell wall in response to pathogen infection to strengthen the plant cell wall and restrict their entry [73,74]. Augmented callose formation is an important feature of β-aminobutiric acid (BABA)-induced resistance against pathogenic fungi that leads to plant protection [75]. Regarding peptide-triggered responses, Pep1, PIP1, and SCOOP12 were reported to induce the accumulation of callose in Arabidopsis plants although to a much lesser extent than flagellin or chitin [14,16]. 

Stomatal closure is also among the inducible defenses triggered by plants under attack since stomata are sites of bacterial pathogen entry in the plant [76]. In this regard, PIP1 was found to induce stomatal closure in Arabidopsis [14]. On the other hand, PNP-A was also reported to regulate stomatal closure upon biotic stress since the pnp-A mutant displayed reduced stomatal closure and higher SA-related responses to bacterial infection, while a PNP-A-overexpressing line closed its stomata more efficiently and lowered SA responses [22]. Interestingly, pnp-A displayed higher resistance against *Pseudomonas syringae* pv *tomato* DC3000 while the overexpression of PNP-A showed higher susceptibility, agreeing with the negative relation with SA-responsive defenses, but not with the ability to regulate the stomatal closure. In this study, the authors infiltrated the bacteria, thus overpassing the defense mediated by the stomata closure. Indeed, Ficarra et al. (2018) [77] used the opposite phenotype, and when using surface inoculation of the bacteria, the bacterium had to first deal with stomatal immunity. 

Finally, sometimes plants can induce indirect defenses upon stimuli perception that includes the release of volatile organic compounds (VOCs) to attract pest natural enemies. Additionally, the released VOCs also prime distal parts of the plant or alerts neighbor plants of upcoming stress. In maize, ZmPep3 treatment triggered an enhanced emission of volatiles, which included terpenes and shikimate pathway-derived compounds that made plants more attractive to lepidopteran herbivore parasitoids [19]. In tomato, Systemin induces the emission of volatiles that, on the one hand, attract pest natural enemies and, on the other hand, alert neighboring plants priming their defenses [78,79].

### 2.3. Role of Phytocytokines in the Defense Response of Peptide-Induced Resistancegainst Pests and Pathogens

Several studies have demonstrated that changing endogenous levels of some phytocytokines by overexpressing or silencing the precursor peptide produces changes in the natural resistance of plants against different attackers confirming their key role in plant defense (Table 2).

Constitutive overexpression of the Pep1 precursor PROPEP1 confers resistance to the root pathogen *Pythium irregulare* in Arabidopsis [11]. Similarly, overexpression of prePIP1 and prePIP2 in Arabidopsis induces resistance against *P. syringae Pst* DC3000 and Foc 699 [14]. In the same line, overexpression lines of the CEP4 precursor displayed enhanced resistance against *Pseudomonas syringae* pv. tomato (Pto), whereas loss-of-function mutants showed susceptibility against the same pathogen [27]. In tomato, Prosystemin-overexpressing plants are more resistant to several attackers including aphids, larvae, and necrotrophic fungi [80], as well as plant viruses [81]. HypSys overexpression in tobacco leads to enhanced resistance to *Helicoverpa armigera* larvae [82]. In contrast, plants expressing antisense ProSystemin were more susceptible to *Manduca sexta* larvae [83]. A knockout mutant of SCOOP12 precursor showed higher susceptibility to *Erwinia amylovora* but enhanced resistance to *Alternaria brassicicola* [16]. Seemingly, loss of PSK signaling reduces resistance against necrotrophic fungi [84], whereas, at the same time, it increases resistance to biotrophic bacteria [85] and fungi [34]. Another example is the GRI peptide that triggers an increase in cell death and increases the resistance to virulent bacteria [25]. 

A contrasting effect on the resistance to biotrophic and necrotrophic pathogens is observed among phytocytokines. This indicates specific roles of plant phytocytokines in resistance according to the attacker’s lifestyle and might be correlated with the specific hormonal regulation upon phytocytokine perception. Thus, it makes sense that peptides involved in defense against herbivores may also defend against necrotrophs since both defense responses usually involve JA regulation. For instance, Systemin is effective against several types of herbivores, such as caterpillars and aphids, as well as against necrotrophic fungi such as *B. cinerea* [80], whereas, although not tested, it is likely not involved in defense against hemibiotrophic such as *P. syringae*. Similarly, in Arabidopsis, PNP-A was shown to antagonize SA-mediated and SA-primed defenses, thus the overexpression of PNP-A resulted in compromised resistance to *Pst* DC3000 [22]. 

**Table 2:** Effect of overexpression of phytocytokines or their precursors.

Plan Species of Origin	Peptide/Precursor	Recipient Plant/Organism	Effect	References
Arabidopsis	PROPEP1	Arabidopsis	Resistance to *Pythium irregulare* and *Pseudomonas syringae*	[11]
Arabidopsis	PrePIP1	Arabidopsis	Resistance to *foc 699*	[14]
Arabidopsis	SCOOP	Arabidopsis	Resistance to *Alternaria brassicicola*	[16]
			Susceptibility against *E. amylovora*	[21]
Arabidopsis	RALF23	Arabidopsis	Susceptibility to *Pto* DC3000 COR and *P. cucumerina*	[23]
Arabidopsis	IDL6	Arabidopsis	Susceptibility to *P. syringae Pst* DC3000	[24]
Arabidopsis	GRI	Arabidopsis	Susceptibility to *P. syringae Pst DC3000*	[25,26]
Arabidopsis	CEP4	Arabidopsis	Resistance to *P. syringae* Pto	[27]
Tomato	ProSystemin	Tomato	Resistance to herbivore	[80]
			Resistance to aphids	[80]
			Resistance to B. cinerea and *A. alternata*	[80]
			Reduced susceptibility to *Cucumber mosaic virus*	[81]
Tomato	ProSystemin	Arabidopsis	Resistance to *B. cinerea*	[86]
Tomato	PSK	Arabidopsis	Susceptibility to *Fusarium oxysporum*	[34]
Arabidopsis	PSK	Tomato	*Botrytis cinerea*	[35]
Maize	Zip1	*Ustilago maydis*	Resistance against *Ustilago maydis*	[37]
Tobacco	HypSys	Tobacco	Resistance to *Helicovera armigera*	[82]

## 3. Phytocytokines/Peptides in Plant-Induced Resistance and Priming

In addition to direct responses to a challenge, plants also evolved the ability to activate stronger defense by inducing resistance mechanisms (IR) at local and distal plant tissues through the so-called systemic induced resistance (ISR) [87]. The state of IR can be achieved by exposing plants to biological organisms but also by treating plants with proteins, xenobiotics, natural extracts, DNA, VOCs, physical damage, or chemicals [56,75,87,88,89]. Plants in the IR state show augmented defense responses and better performance upon different challenges [56,87,90,91]). Moreover, plants expressing IR trigger specific short-term defense mechanisms [88] and activate chromatin remodeling providing a longer-term “plant memory” [92]. When the plant’s perception of an IR stimulus does not trigger major changes in the plant metabolism directly but rather shows an augmented response only when the challenge appears, it is known as “defense priming” [56,75,93]. Primed plants exhibit a faster and stronger defense response that leads to enhanced disease protection against a broad range of pathogens and is associated with low fitness cost [93,94,95]. Recently, there has been a consensus that the IR phenotype is a sum of both direct and primed defense activation [87]. Priming during SAR and ISR is expressed in distal tissues upon the perception of the secondary challenge [56]. At times, the same stimuli can trigger either direct induced resistance or primed defenses depending on the concentration [75]. This indicates the importance of establishing an optimal dose threshold for achieving beneficial effects when using a resistance elicitor. 

The biological function of small, secreted peptides in plant basal immunity has been extensively studied. In the above-mentioned sections, we describe endogenous phytocytokines triggering a huge range of defense responses and signaling cascades upon cell damage by pests and pathogens to amplify the defense response (Figure 1A). Similar responses are observed when their precursor is overexpressed. However, what is the outcome of their exogenous application against an upcoming attack? Because of their ability to activate the plant immune system and induce defensive responses at very low concentrations, they can be considered suitable candidates as defense elicitors (Figure 1B). 

In natural environments, phytocytokines are released after plant perception of a biotic challenge during the activation of the first layer of immune responses, PTI. Then phytocytokines bind to their receptors to amplify and strengthen the already-activated defenses and spread the danger alarm to adjacent cells (Figure 1A). Hence, as common defense strategies, cellular responses are activated when both the phytocytokine and the challenge of PAMP are present. In contrast, when peptides are used as defense elicitors, the plant perceives the phytocytokine prior to the challenge. The plant recognizes them as danger signals and activates moderate defense signaling, thus when a future biotic challenge occurs, the plant poses an enhanced defensive response displaying peptide-IR (Figure 1B). However, the effect of exogenously applied peptides in the plant’s defensive responses may differ from that triggered naturally when the endogenous peptide is released after the challenge.

### 3.1. Peptide-Induced Resistance against Pests and Pathogens

Although the natural function of phytocytokines is currently under study, their potential for induced resistance when applied exogenously needs further research. Nevertheless, there is some promising evidence of their benefits on plant effects (Table 3).

Exogenous treatment of Pep3, PIP1, or SCOOP12 leads to Arabidopsis resistance to *Pst* DC 3000 [9,14,16]. In maize, the ZmPep1 treatment confers resistance to the necrotrophic fungal pathogens *Cochliobolis heterostrophus* and *Colletotrichum graminicola* [12], whereas ZmPep3 treatment produced a reduction in *Spodoptera exigua* larval growth [19]. In tomato, CAPE1 application induces resistance to both the herbivore *Spodoptera litura* and the biotrophic pathogen *P. syringae* DC3000 [15]. Similarly, the exogenously applied phytosulfokine (PSK), as well as Systemin, enhances resistance to the necrotrophic fungus *B. cinerea* in tomato [35,67]. In addition, Systemin treatment also impairs the larval growth of *Spodoptera litoralis* [67]. SAMPs produced by citrus resistant to *Candidatus liberibacter* asiaticus induce systemic resistance against this bacterium when sprayed on the leaves of HLB-sensitive cultivars [17]. Hence, it seems clear that peptides induce resistance against herbivores and necrotrophs.

Seemingly, compared to endogenous peptides, exogenous treatments trigger specific defense pathways inducing resistance against pathogens with the same lifestyle. In this line of evidence, when alternative pathogens with opposite lifestyles infect the plant, pretreatment with peptides may trigger susceptibility. This is the case for Zip1 and PNP-A treatments that trigger susceptibility against *B. cinerea* and *Pst* DC3000, respectively [22,37].

### 3.2. Cross-Species Perception and Peptide-Induced Resistance

Interestingly, a few studies have reported heterologous peptide sensing and signaling in taxonomically distant plant species. Although a report claims that tobacco cells do not respond to exogenous Systemin treatment [97], a later study showed that tobacco calli and suspension cells responded to Systemin by both MAPK activation and weak-medium alkalinization [98]. In addition, constitutive expression of the tomato ProSystemin gene in tobacco considerably affected the plant metabolism by inducing the synthesis of host proteins, several of which are involved in protection against pathogens, suggesting the ability of tobacco to reproduce Systemin signaling [99]. More surprisingly, Zhang et al. (2017) [86] reported that tomato Systemin was sensed by Arabidopsis plants, leading to an inhibition of seedling root growth and the expression of the plant defensin PDF1.2. On the other hand, tobacco cells transformed with the Arabidopsis Pep1 receptor PEPR1 responded to nanomolar concentrations of Pep1, producing a strong alkalinization of the cell culture medium, again suggesting the capacity of tobacco to activate signaling upon heterologous peptide treatment [47]. Later, Huffaker and coworkers (2013) [19] found ZmPep orthologs in rice (OsPep2) and sorghum (SbPep1) and tested their ability to induce volatile emissions in maize plants. They found that both peptides elicited a full spectrum of herbivore-associated volatiles at the same level as those induced by maize Peps. This suggests that Peps from rice and sorghum species might be able to induce resistance in maize similarly to ZmPeps. 

However, evidence of peptide-induced resistance in heterologous species is very scarce (Table 3). Heterologous peptides, including Sytemins from *Solanaceae* and AFPs from radish, confer resistance to the necrotroph *Plectosphaerella cucumerina* in Arabidopsis [96]. In addition, very recently, it was demonstrated that Systemin is also able to induce resistance against necrotrophic fungi in the taxonomically distant species *Vitis vinifera*, as well as in *Solanum melongea*, which is taxonomically closer but still does not produce the peptide [29]. The functionality of peptide treatments in cross-species IR is emerging as a very interesting tool to be used as general agents of biocontrol and thus deserves further research.

## 4. Cooperative Functioning of Peptides in Innate Immunity and Induced Resistance

As previously mentioned, defense peptides function as amplifiers of the “warning alarm”. The increasing number of identified peptides functioning as phytocytokines within the same plant species such as Arabidopsis, maize, or tomato suggests a possible interaction between them to coordinate the immune response. Interesting studies indicate a complex network of interconnected peptides in the plant response to stress and defense by performing an in silico analysis of the predicted peptide interactome [100].

There is evidence of peptide cooperation to amplify the defense response, and PIP-RLK7 and PEP1-PEPR1 cooperate by amplifying the immune response triggered by the PAMP flagellin in Arabidopsis [14]. Similarly, SCOOP12 and Pep1 induce the expression of several of the SCOOP precursors genes, PROSCOOPs, [16], suggesting that Pep1 is cooperating with SCOOPs to amplify its feedback loop. In tomato, Systemin and HypSys function together in the regulation of the long-distance wound signaling response in tomato through the upregulation of the octadecanoid pathway and the synthesis of jasmonates [101]. Finally, CAPE1 is among the signals induced upon wounding plus MeJA treatment together with Systemin, both with a similar expression pattern, which means that both peptides regulate the response to the same stress [15]. All these findings suggest synergistic effects between specific peptides, raising the question of the possible coapplication of different peptides or peptides plus other danger signals as an interesting strategy to potentiate Peptide-IR.

In this regard, evidence of the interdependence of PAMP and DAMP signaling has already been reported in the literature. A functional Pep/PEPR1 system is required for complete FLS2 immune signaling, including flg22-induced Ca^2+^ increase, H_2_O_2_ production, defense gene activation, and flg22/FLS2-induced hampering of pathogen growth, whereas the loss of FLS2 similarly impaired PEPR1 signaling [58]. Later, Ma and coworkers (2013) [9] also observed that maximal H_2_O_2_ and NO production in response to Pep3 required the presence of both PEPR1 and FLS2 receptors, again suggesting cooperation between flg22 and Peps signaling.

In terms of IR, a beneficial effect of the coapplication of PAMPs and DAMPs has been described, although it is poorly explored. Klauser et al. (2013) [102] observed that Pep1 triggered oxidative burst when applied to leaf discs and was enhanced by a subsequent application of either Flg22 or chitin. Recently, Pastor et al. (2022) [103] demonstrated that the simultaneous perception of Flg22 (non-self) and DAMPs (self) produces an amplification of PTI, as well as the production of phytocytokines. Similarly, integration between HAMP and DAMP signaling was evidenced since the application of a rice Pep3 together with insect oral secretions produced an amplification of a great variety of defense responses in rice plants, such as the activation of MAPK and the production of defense hormones and metabolites [104].

The previous observations demonstrate that the co-application of danger signals of different natures could be a useful tool to enhance the IR defensive response, mediated by phytocytokines.

## 5. Conclusions and Future Perspective

Phytocytokines are a type of small molecule that is present in plants when stress appears. Much work is still to be performed to decipher the role of these molecules in cell-to-cell communication or their induction of downstream cell events. Being small molecules, processing can be very complex as already relayed throughout this review, and it is of great interest to clarify each step in the production of the peptide and its role in defense against fungi, bacteria, viruses, nematodes, and herbivores. Studies at both molecular and applied levels are necessary to fully explore the action of the phytocytokines, including their precursors. The plant application or co-application of these small molecules will also allow us to explore the possibilities of adaptive immunity against biotic stress and crop protection using natural compounds that exert an effect on plants. In this regard, it may also be interesting to follow the entire biological cycle of the plant to determine when the best moment is for application and the impact on yield production.

## Figures and Tables

**Table 1:** Main features of phytocytokines in biotic stress.

Phytocytokine	Species of Origin	Signal Transduction	Induced Defense Responses and Signaling	References
Peps	Arabidopsis	PEPR1 and PEPR2	media alkalinization, H_2_O_2_ *PDF1.2* and PROPEPs expression	[11]
		BAK1	Ca^2+,^ ET, callose	[9]
		BIK1/PBL1	Ca^2+^, H_2_O_2_ NO *MPK3* and *WRKY33* expression	[18]
ZmPep1	Maize		JA, ET, defense gene expression, defense metabolites accumulation	[12]
ZmPep3	Maize		JA, ET, defense gene expression, volatiles emission, phytoalexin	[19]
PIP1	Arabidopsis	RLK7	ROS, *FRK1*, *WRKY30*, *WRKY33*, *WRKY53*, *MYB51* and *PR1* expression	[14]
		partially BAK1- dependent	MAPK, Callose, Stomatal closure	
SCOOP12	Arabidopsis	MIK2-BAK1/SERK4	ROS, callose	[16]
			Phosphatidic acid (PA) *FRK1* expression	[20]
SCOOPs		BIK1/PBL1	Ca^2+^, ROS, MAPK Ethylene, defense gene expression	[21]
PNP-A	Arabidopsis	PNP-R2	antagonizes SA responses, stomatal closure	[22]
RALF23	Arabidopsis	FER-BAK1	Ca^2+^, Media alkalinization	[23]
			Antagonizes PAMP-induced ROS	
IDL6	Arabidopsis	HAE and HSL2	Poligalacturonase gene ADPG2	[24]
GRI	Arabidopsis	PRK5	ROS-dependent Cell death, hormones	[25,26]
CEP4	Arabidopsis	CEPR1/2 and RLK7	Ca^2+^, MAPK Ethylene, FRK1 expression	[27]
Systemin	Tomato	SYR1	Opening of ion channels, Ca^2+^, MAPKs JA, defense genes	[10]
		SYR2	CDPKs, ROS Protease inhibitors	[28]
		PORK1	CAT and APX activity Volatiles emission	[29]
PotSys1 and 2	Potato	SYR1 and SYR2	Proteinase inhibitors	[30]
PepSys	Pepper			
NishSys	Nightshade			
HypSys1, 2 and 3	Tomato		Media alkalinization JA, PI-I, and PI-II	[31]
	Potato		H_2_O_2_ PIs, JA, defense-related genes, antioxidant defensive enzymes	[32]
TobHypSys 1 and 2	tobacco		Media alkalinization, MAPK Proteinase inhibitors	[33]
CAPE1	Tomato		H_2_O_2_ SA, defense gene expression	[15]
PSK	Arabidopsis	PSRKs	Ca^2+^ IAA and Auxin-dependent responses	[34]
	Tomato			[35]
PSY1	Arabidopsis	PSY1R		[34]
SubPep	Soybean		Media alkalinization Chitinase1b, CYP93A1, chalcone synthase and PDR12 gene expression	[36]
Pep914	Soybean		Media alkalinization CYP93A1, Chib1-1, and chalcone synthase gene expression	[13]
Pep890				
Zip1	Maize		SA, SA, and JA marker genes, defense-related genes	[37]
SAMP	Citrus		Defense gene expression	[17]

**Table 3:** Effects of exogenous peptide applications in resistance against pest and pathogens.

Plant Species of Origin	Peptide	Recipient Plant	Effect	References
Arabidopsis	Pep3	Arabidopsis	Resistance to *Pst DC 3000*	[9]
Arabidopsis	PIP1	Arabidopsis	Resistance to *Pst DC 3000*	[14]
Arabidopsis	SCOOP12	Arabidopsis	Resistance to *Pst DC 3000*	[16]
Maize	ZmPep1	Maize	Resistance to *Cochliobolis heterostrophus* and *C. graminicola*	[12]
Maize	ZmPep3	Maize	Resistance to *Spodoptera exigua*	[19]
Tomato	CAPE1	Tomato	Resistance to *Spodoptera litura*	[15]
			Resistance to *Pst DC 3000*	
Tomato	PSK	Tomato	Resistance to *B. cinerea*	[35]
Tomato	Systemin	Tomato	Resistance to *Spodoptera litoralis*	[67]
			Resistance to *B. cinerea*	
Arabidopsis	PNP-A	Arabidopsis	Susceptibility to *P. syringae*	[22]
Maize	Zip1	Maize	Susceptibility to *B. cinerea*	[37]
Tomato	Systemin	Arabidopsis	Resistance to *P. cucumerina*	[96]
Potato	PotSysII			
Pepper	PepSys			
Nightshade	Nishsys			
Tomato	HypSys			
Radish	AFP’s			
Arabidopsis	Pep1	Arabidopsis	Resistance to *P. cucumerina*	[96]
Tomato	Systemin	Eggplant	Resistance to *B. cinerea*	[29]
		*Vitis vinifera*		
Citrus	SAMP	Citrus	Resistance to *Candidatus liberibacter asiaticus*	[17]

## Data Availability

Data sharing no applicable to this article.
